# Out-of-pocket payments and economic consequences from tuberculosis care in eastern China: income inequality

**DOI:** 10.1186/s40249-020-0623-8

**Published:** 2020-02-05

**Authors:** Yan Liu, Cai-Hong Xu, Xiao-Mo Wang, Zhen-Yu Wang, Yan-Hong Wang, Hui Zhang, Li Wang

**Affiliations:** 1 Department of Epidemiology and Biostatistics, Institute of Basic Medical Sciences Chinese Academy of Medical Sciences, School of Basic Medicine Peking Union Medical College, Beijing, 100005 China; 2 Department of Critical Care Medicine, The Second People’s Hospital of Shenzhen & First Affiliated Hospital of Shenzhen University, Health Science Center, Shenzhen, 518035 China; 3 National Center for Tuberculosis Control and Prevention, China Center for Disease Control, Beijing, 100226 China

**Keywords:** Out-of-pocket payment, Tuberculosis, Impoverishment, Coping strategy, Eastern China

## Abstract

**Background:**

Despite the availability of free tuberculosis (TB) diagnosis and treatment, TB care still generates substantial costs that push people into poverty. We investigated out-of-pocket (OOP) payments for TB care and assessed the resulting economic burden and economic consequences for those with varying levels of household income in eastern China.

**Methods:**

A cross-sectional study was conducted among TB patients in the national TB programme networks in eastern China. TB-related direct OOP costs, time loss, and coping strategies were investigated across households in different economic strata. Analysis of Variance was used to examine the differences in various costs, and Kruskal-Wallis tests were used to compare the difference in total costs as a percentage of annual household income.

**Results:**

Among 435 patients, the mean OOP total costs of TB care were USD 2389.5. In the lower-income quartile, OOP payments were lower, but costs as a percentage of reported annual household income were higher. Medical costs and costs prior to treatment accounted for 66.4 and 48.9% of the total costs, respectively. The lower the household income was, the higher the proportion of medical costs to total costs before TB treatment, but the lower the proportion of medical costs patients spent in the intensive phase. TB care caused 25.8% of TB-affected households to fall below the poverty line and caused the poverty gap (PG) to increase by United States Dollar (USD) 145.6. Patients in the poorest households had the highest poverty headcount ratio (70.2%) and PG (USD 236.1), but those in moderately poor households had the largest increase in the poverty headcount ratio (36.2%) and PG (USD 177.8) due to TB care. Patients from poor households were more likely to borrow money to cope with the costs of TB care; however, there were fewer social consequences, except for food insecurity, in poor households.

**Conclusions:**

Medical and pretreatment costs lead to high costs of TB care, especially among patients from the poorest households. It is necessary to train health system staff in general hospitals to promptly identify and refer TB patients. Pro-poor programmes are also needed to protect TB patients from the medical poverty trap.

## Background

Tuberculosis (TB) remains a serious threat to global public health, ranking as the leading cause of death from infectious diseases worldwide [1]. According to a World Health Organization (WHO) [2] report in 2018, 10 million people were estimated to develop TB in 2017, and cases in China accounted for 9% of the new cases.

TB does not homogeneously affect people at different economic levels. First, poor people are at greater risk of TB infection, comorbidity, and poor treatment outcomes due to poor living conditions and financial difficulties, among others [3–9]. Second, TB leads to a severe economic burden for patients and their households by affecting productive labour [8, 10, 11]. Despite the international norm for “free TB care” policies, these policies cover only some diagnostic tests and first-line anti-tuberculosis drugs during TB treatment [12]. TB patients still face great economic burden due to a considerable amount of out-of-pocket (OOP) medical expenses, high costs of travel and food, and income loss before and during treatment [12, 13]. Finally, TB has implications for patients and their households in terms of impoverishment, pushing them below the poverty line. Low-income people often lack savings and income, and the substantial costs of TB care compel them to use coping strategies, such as borrowing and selling to pay for TB care [14], making them even poorer [11, 15].

In China, healthcare providers in general hospitals are required to refer suspected TB patients to local designated TB facilities in the national TB programme (NTP) networks. TB patients diagnosed in NTP networks are provided with one chest X-ray and three sputum smears at the time of diagnosis and free first-line anti-tuberculosis drugs in the standard chemotherapy regimen. However, studies have shown that there is still a severe economic burden associated with TB diagnosis and treatment under this policy in China, including costs due to delayed diagnosis, drugs and tests beyond the standard treatment regimen [16, 17]. Additionally, TB affects the productive labour of the household, which aggravates the household economic burden. Thus, it is necessary to evaluate the economic impacts associated with TB care at the household level to better inform health policy. However, only a limited number of studies have analysed TB care costs at the household level in China [18].

Conducting a cross-sectional survey in eastern China, this study aimed to comprehensively describe OOP payments and the economic consequences for TB care at the household level. Our results have implications for potential policies to reduce TB-related poverty and increase equity in accessing care.

## Methods

### Setting

Eastern China is the most economically developed area in China, with an estimated 0.7 billion people from 11 provinces. Although recent data showed a decreasing incidence of TB in eastern China, 260 758 new cases have been reported in the TB Management Information System in 2016.

### Study design and participants

This survey was adapted from the WHO [19] protocol. A cross-sectional study design with retrospective data collection was used. The study population included all patients who received TB treatment within NTP networks in 2016–2017 in eastern China. All patients (including drug-susceptible TB and multidrug-resistant TB patients) were aged ≥ 15 years and had been treated in the intensive phase or continuation phase for at least 2 weeks.

### Sampling methodology and sample size estimation

A multi-stage stratified cluster sampling method was used [19]. The cluster was defined as the basic management unit (BMU) in NTP networks. We used the proportion of families experiencing catastrophic costs due to TB to estimate the sample size. Based on an assumption that 40% of families experienced catastrophic costs, a relative precision of 0.25, an average cluster size of 50, a between-cluster variation coefficient of 0.32, and an α of 0.05, the required sample size was estimated to be 399 with sampling from eight clusters. The sampling procedures were as follows. First, two provinces were selected from the 11 provinces in eastern China. Second, all BMUs in the selected provinces were listed and stratified into urban or rural areas. Cities and municipalities were defined as urban areas, and counties were defined as rural areas. The number of BMUs in urban and rural areas was determined by the probability proportionate to the sample size based on TB notifications in 2015. One and three BMUs were selected in each urban and rural area, for a total of eight clusters. Fifty consecutive TB patients (including new and relapsed patients) were expected to be selected from each BMU, and 435 TB patients were enrolled finally.

### Data collection

Quantitative methods were used to collect the data. An interview-administered structured questionnaire for TB patients was conducted between April and June 2017. Twelve medical post-graduate students and three faculty members from medical universities were trained as interviewers. Additionally, at each study site, 4–6 local volunteers who were familiar with the local customs, social situations and local languages were invited to help administer the questionnaires. All volunteers were trained to understand the questionnaire well. In the survey, if necessary (e.g., when interviewing patients with lower levels of education or who spoke local languages, etc.), these volunteers helped to communicate with patients to collect the data. All the interviewers, volunteers, and patients were Han. There were no cultural differences among them. The following information was obtained from TB patient interviews and treatment cards: (1) demographic and socio-economic information (age, sex, education, occupation, and household income, etc.); (2) direct medical OOP costs, non-medical OOP costs per visit, TB patient or guardian time loss, and coping strategies (loans and sale of assets) due to TB health care or hospitalization from the time of the self-reported onset of TB-related symptoms until the treatment status at interview; and (3) TB-related diagnosis (diagnosis date and place and type of TB) and treatment (treatment regimen, total duration of planned treatment, and current treatment phase).

### Cost measurement and extrapolation

The operational definitions of the key study variables (TB treatment phases and TB costs) are summarized in Table 1.

**Table 1:** Glossary of the operational definitions of TB treatment and costs

Before TB treatment	Between symptom onset and treatment initiation
During TB treatment	From treatment initiation up to treatment completion
Entire episode of TB	Before TB treatment and the treatment phases combined
Direct medical costs	OOP costs of medical examinations and medicines linked to TB diagnosis and treatment incurred after any reimbursements made to patients
Direct non-medical costs	Costs for transport, accommodation, food expenditures, and nutrition supplements due to TB
Direct costs	Direct medical + direct non-medical costs
Indirect costs	Patients’ and guardians’ lost income due to TB-related time off work during the TB episode, which was estimated using the time off work multiplied by the reported individual income prior to the onset of TB
Total costs	Direct + indirect costs

Abbreviations: *TB* Tuberculosis, *OOP* Out-of-pocket

Because we collected the costs and time loss only from the self-reported onset of symptom until the treatment status at interview, the costs, visit time, and time loss from the time of the interview to the end of the expected continuation phase were extrapolated. We extrapolated the costs according to the internationally defined duration of the intensive and continuation phases: (1) two months for the intensive phase and four months for the continuation phase for new patients; (2) two and six months, respectively, for relapsed patients; and (3) six and six months, respectively, for multi-drug resistant TB treatment. For patients who interviewed during the continuation phase, their past costs and treatment time during the continuation phase were used to extrapolate the expected costs for the whole continuation phase with a generalized linear model. For patients interviewed in the intensive phase, we first extrapolated their complete intensive costs using a generalized linear model. Then, we estimated their costs during the continuation phase based on the patients who were interviewed in the continuation phase in the same region by adjusting for age, gender, medical insurance, educational level, family breadwinner status, hospitalization, and comorbidity.

### Household income quartiles

TB patients were divided into four equally sized groups or quartiles (Q1 to Q4) based on their household income. The lowest and highest 25% of households were defined as the poorest (Q1) and the richest families (Q4).

### Measuring impoverishment due to OOP payments

The headcount (HC) index was used to measure the proportion of TB patients who were poor due to OOP payments for TB care. The pre-payment headcount was based on per capita income before TB diagnosis, while post-payment headcount was based on per capita income after TB diagnosis. x_i_ was defined as individual i’s annual household’s per capita income, and *Z* was the poverty line at each survey site. Then, P_i_ was defined as 1 if x_i_ < *Z* and as 0 otherwise. The HC [20] was:

1 $$ \mathrm{HC}=\frac{1}{N}\sum \limits_{i=1}^N{\mathrm{P}}_i $$

where *N* was the sample size.

The poverty gap (PG) was defined as the average of all shortfalls from the poverty line. g_i_ equalled x_i_-*Z* if x_i_ < *Z*, and zero otherwise. The pre-payment PG was based on per capita income before TB, while the post-payment PG was based on per capita income after TB. The average PG was:

2 $$ \mathrm{PG}=\frac{1}{N}\sum \limits_{i=1}^N{\mathrm{g}}_i $$

where *N* was the sample size.

## Data analysis

The statistical package SAS 9.4 (Windows, SAS Institute, Cary, North Carolina, USA) was used to analyse the data. Costs, including total costs, direct costs, and indirect costs, were presented as their arithmetic means whether the data were Gaussian or non-Gaussian because this approach is considered to be robust for health economics data analysis [15–17]. All costs and incomes were estimated in United States Dollars (USD) (based on a currency exchange rate of Chinese Yuan (CNY) 675 to USD 100 in 2017).

The categorical data were summarized as proportions, and *χ*^2^ tests were used to test the differences. Analysis of Variance was used to examine the differences in various costs, and Kruskal-Wallis tests were used to compare the difference in total costs as a percentage of annual household income.

## Results

### Socio-demographic characteristics

A total of 435 individuals with TB from eight BMUs in NTP networks in eastern China were enrolled. Most individuals were male (75.2%), were of working age (38.9% in the 40–60 year range), had educational levels lower than high school (83.0%), and lived in rural areas (74.9%). Nearly all individuals had health insurance, and 62.9% were the primary breadwinners for their households. Most of the patients did not been tested for their HIV statuses, and less than 1% of them reported HIV positive. Two-thirds of the patients were smear-negative, and 90.8% were newly diagnosed. More patients from poor households than those from higher-income households were from rural areas and had lower educational levels (Table 2).

**Table 2:** Socio-demographic and clinical characteristics of the participants

Variable	Total (*n* = 435)	Income quartiles^a^				*P*-value
		Q1 (*n* = 104)	Q2 (*n* = 113)	Q3 (*n* = 109)	Q4 (*n* = 109)	
Age group in years (%)						< 0.001
< 40	118 (27.1)	7 (6.7)	31 (27.4)	40 (36.7)	40 (36.7)	
40–60	169 (38.9)	43 (41.3)	51 (45.1)	36 (33.0)	39 (35.8)	
≥ 60	148 (34.0)	54 (51.9)	31 (27.4)	33 (30.3)	30 (27.5)	
Male (%)	327 (75.2)	83 (79.8)	82 (72.6)	77 (70.6)	85 (78.0)	0.352
Education level (%)						0.011
Illiterate or semi-illiterate	89 (20.5)	29 (27.9)	17 (15.0)	25 (22.9)	18 (16.5)	
Primary school	112 (25.7)	30 (28.8)	31 (27.4)	21 (19.3)	30 (27.5)	
Middle school	160 (36.8)	37 (35.6)	43 (38.1)	47 (43.1)	33 (30.3)	
High school	49 (11.3)	8 (7.7)	16 (14.2)	9 (8.3)	16 (14.7)	
College and above	25 (5.7)	0 (0.0)	6 (5.3)	7 (6.4)	12 (11.0)	
Residence (%)						0.005
Urban	109 (25.1)	16 (15.4)	23 (20.4)	33 (30.3)	37 (33.9)	
Rural	326 (74.9)	88 (84.6)	90 (79.6)	76 (69.7)	72 (66.1)	
Primary breadwinner (%)						0.341
Yes	273 (62.9)	72 (69.2)	72 (64.3)	66 (60.6)	63 (57.8)	
No	161 (37.1)	32 (30.8)	40 (35.7)	43 (39.4)	46 (42.2)	
Marital status (%)						0.001
Unmarried	68 (15.6)	9 (8.7)	17 (15.0)	24 (22.0)	18 (16.5)	
Married	310 (71.3)	76 (73.1)	73 (64.6)	76 (69.7)	85 (78.0)	
Divorced	24 (5.5)	10 (9.6)	12 (10.6)	1 (0.9)	1 (0.9)	
Widowed	33 (7.6)	9 (8.7)	11 (9.7)	8 (7.3)	5 (4.6)	
Place of registration (%)						<0.001
Integrated hospital	283 (65.1)	35 (33.7)	66 (58.4)	84 (77.1)	98 (89.9)	
Local CDC	152 (34.9)	69 (66.3)	47 (41.6)	25 (22.9)	11 (10.1)	
Insurance (%)						0.002
UEBMI	31 (7.1)	1 (1.0)	5 (4.4)	11 (10.1)	14 (12.8)	
URBMI	43 (9.9)	4 (3.8)	9 (8.0)	16 (14.7)	14 (12.8)	
NCMS	338 (77.7)	94 (90.4)	91 (80.5)	78 (71.6)	75 (68.8)	
Others	23 (5.2)	5 (4.8)	8 (7.1)	4 (3.7)	6 (5.5)	
TB category (%)						0.626
New case	395 (90.8)	94 (90.4)	100 (88.5)	102 (93.6)	99 (90.8)	
Relapse case	40 (9.2)	10 (9.6)	13 (11.5)	7 (6.4)	10 (9.2)	
Sputum status at diagnosis (%)						0.053
Undetected	16 (3.8)	3 (2.9)	6 (5.6)	0 (0.0)	7 (6.5)	
Positive	132 (31.1)	27 (26.0)	30 (27.8)	36 (33.6)	39 (36.1)	
Negative	277 (65.2)	72 (69.2)	72 (66.7)	71 (66.4)	62 (57.4)	
HIV (%)						0.569
Positive	1 (0.2)	0 (0.0)	0 (0.0)	0 (0.0)	1 (0.9)	
Negative	97 (22.5)	24 (23.1)	21 (18.6)	23 (21.1)	29 (26.6)	
Unknown	333 (77.3)	79 (76.0)	89 (78.8)	86 (78.9)	79 (72.5)	
Comorbidity^b^ (%)						0.226
Yes	274 (62.9)	50 (48.1)	39 (34.5)	42 (38.5)	43 (39.4)	
No	161 (37.1)	54 (51.9)	74 (65.5)	67 (61.5)	66 (60.6)	

^a^Income quartiles are arranged from lower to higher (Q1 = lower; Q4 = higher)

^b^Comorbidity indicates TB with one or more of the following diseases: diabetes, chronic liver diseases, chronic renal disease, anaemia, hypertension and other chronic disease.

Abbreviations: *CDC* Centers for Disease Control, *UEBMI* Urban Employee Basic Medical Insurance, *URBMI* Urban Residents’ Basic Medical Insurance, *NCMS* New Cooperative Medical System, *Q1* 1st Quartiles, *Q2* 2nd Quartiles, *Q3* 3rd Quartiles, *Q4* 4th Quartiles

### Costs of TB

**Fig. 1:**
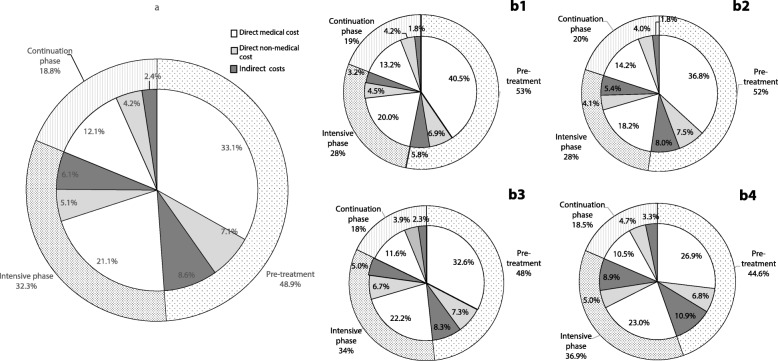
Distributions of OOP payments across the household income quartiles. (a) Distribution of OOP payments in the total population. (b1) Distribution of OOP payments in Q1 households. (b2) Distribution of OOP payments in Q2 households. (b3) Distribution of OOP payments in Q3 households. (b4) Distribution of OOP payments in Q4 households. Abbreviations: OOP: Out-of-pocket; Q1: 1st Quartiles; Q2: 2nd Quartiles; Q3: 3rd Quartiles; Q4: 4th Quartiles

The distribution of TB-related costs is shown in Table 3 and Fig. 1. The average total OOP costs for the entire TB episode were USD 2389.5. Direct costs were much higher than indirect costs, making up the greatest proportion of total costs (82.9%). For direct costs, medical costs were significantly higher than non-medical costs. The lower the household income was, the lower the total costs and component costs. However, the proportion of direct medical costs to total costs was lower for those from higher-income households (60.4%) than for those from the lower-income households (73.7%) (*P* < 0.001).

**Table 3:** Distribution of OOP tuberculosis-related payments (USD) ^a^ across the household income quartiles

Indicators	Total	Income quartiles ^b^				*P*-value
		Q1	Q2	Q3	Q4	
Cost category						
A1. Direct medical costs	1586.7	1305.8	1506.2	1624.7	1900.2	0.046
A2. Direct non-medical costs	393.6	275.7	338.9	439.9	516.6	0.020
Transport	65.1	56.7	67.8	65.9	70.8	0.749
Food	198.6	142.0	178.6	230.0	241.9	0.194
Accommodation	46.9	34.4	45.7	45.1	61.9	0.360
Nutritional supplement	82.7	42.5	46.8	99.0	142.0	0.014
A. Direct costs	1980.3	1581.6	1845.1	2064.6	2416.8	0.046
B. Indirect costs	409.2	189.4	332.2	380.5	727.4	< 0.001
C. Total costs	2389.5	1771.0	2177.3	2445.1	3144.2	< 0.001
TB episode						
E. Pre-treatment	1167.8	941.4	1138.0	1180.5	1401.9	0.307
F. During treatment	1221.8	829.6	1039.3	1264.6	1742.3	0.001
F1. Intensive phase	772.4	490.7	604.2	828.3	1159.7	0.005
F2. Continuation phase	449.4	338.9	435.2	436.3	582.6	0.075

^a^currency exchange rate: CNY 675 to USD 100.

^b^Income quartiles are arranged from lower to higher (Q1 = lower; Q4 = higher).

Note: A = A1 + A2, F = F1 + F2; C = A + B = E + F. Abbreviations: *OOP* Out-of-pocket; *OOP* Out-of-pocket; *USD* United States Dollar; *CNY* Chinese Yuan; *Q1* 1st Quartiles; *Q2* 2nd Quartiles; *Q3* 3rd Quartiles; *Q4* 4th Quartiles

Total costs were similar before treatment and during treatment (48.9% [95% *CI*: 45.8–52.5%] versus 51.1% [95% *CI*: 47.9–54.2%] of total costs, *P* = 0.33). For costs during treatment, total costs in the intensive treatment phase were approximately double those in the continuation treatment phase. Costs incurred before TB treatment showed no difference across household income quartiles; however, the proportion of direct medical costs to total costs before TB treatment was higher in the lower income-quartile households. In contrast, the proportion of direct medical costs to total costs in the intensive phase was higher in the wealthiest households.

### Costs as a percentage of reported annual household income

**Fig. 2:**
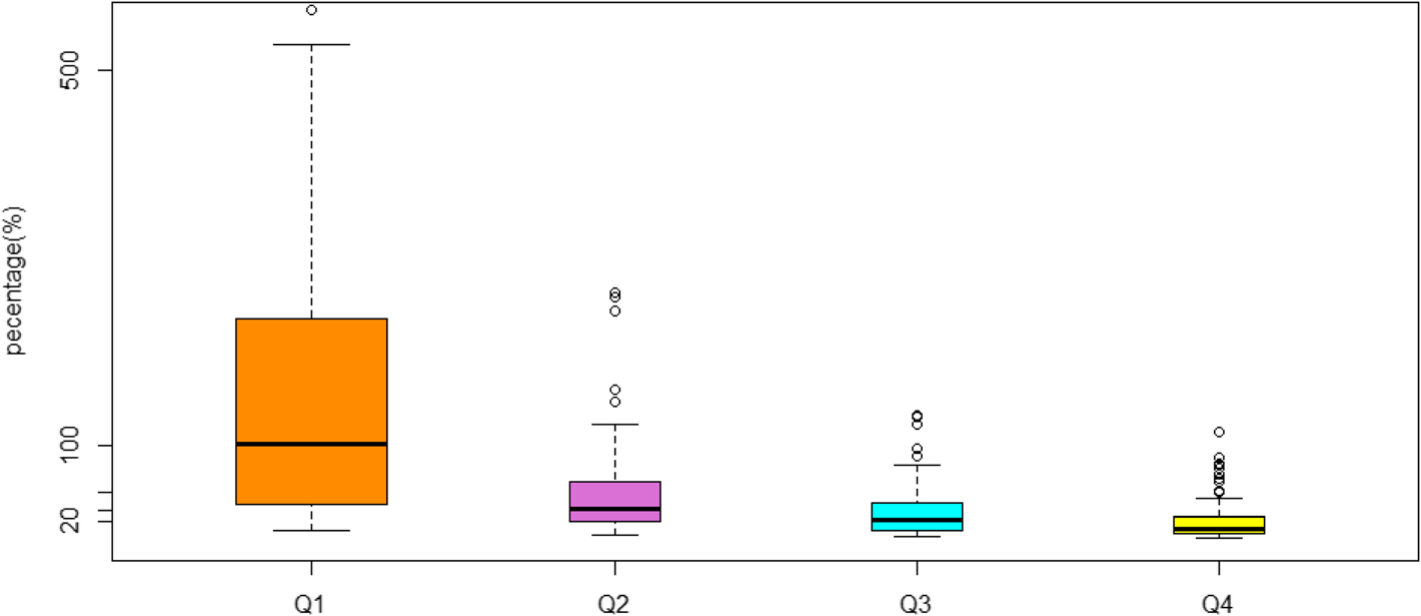
Costs as a percentage of reported annual household income across the household income quartiles. Abbreviations: Q1: 1st Quartiles; Q2: 2nd Quartiles; Q3: 3rd Quartiles; Q4: 4th Quartiles

Total costs were equivalent to 27.4% (IQR: 12.1–64.5%) of the annual household income. Costs as a percentage of reported annual household income were higher (102.0% [IQR: 37.1–235.1%]) in the lower income-quartile households than in the other income quartiles. The total costs were equivalent to 10.5% (IQR: 6.1–23.3%) of the annual household income in the wealthiest households (Q4) (Fig. 2).

### Impoverishment impact of OOP TB-related payments

Before TB diagnosis, poor households (those below the poverty line) accounted for 11.0% of all households, and the PG was USD 26.1. After OOP payments for TB care, 25.8% of non-poor households fell below the poverty line, and PG increased by USD 145.6. The poverty headcount ratio increased to 70.2%, and PG increased to USD 236.1 in the poorest households. We also find that there was a 36.2% increase in the poverty headcount ratio and a USD 177.8 increase in PG due to OOP payments in moderately poor households. Impoverishment due to TB was more common in the poor quartiles (Table 4).

**Table 4:** Impoverishment impact of OOP tuberculosis-related payments

Characteristic	Income quartiles^a^				Total
	Q1	Q2	Q3	Q4	
Poverty headcount (%)					
Pre-payment (a)	43.3	2.7	0.0	0.0	11.0
Post-payment (b)	70.2	38.9	26.6	12.8	36.8
Poverty impact (b)–(a)	26.9	36.2	26.6	12.8	25.8
PG (USD)					
Pre-payment (c)	107.5	1.9	0.0	0.0	26.1
Post-payment (d)	236.1	179.7	137.9	76.6	156.9
Poverty impact (d)–(c)	128.6	177.8	137.9	76.6	145.6

^a^Income quartiles are arranged from lower to higher (Q1 = lower; Q4 = higher). Abbreviations: *Q1* 1st Quartiles; *Q2* 2nd Quartiles; *Q3* 3rd Quartiles; *Q4* 4th Quartiles. *OOP* Out-of-pocket, *USD* United States Dollar

#### Coping strategies and social consequences

Approximately 48.6% of the patients adopted coping strategies to handle the costs of TB. They either borrowed money only (45.8%), sold assets only (0.5%), or did both (2.3%). Compared with individuals from the higher-income quartiles (31.2%), individuals from the lower-income quartiles (66.4%) more commonly borrowed money to cope with the costs of TB care (*P* < 0.001).

Most (78.1%) of the individuals reported that TB affected their personal and social lives, resulting in food insecurity (34.3%), job losses (35.9%), social exclusion (31.9%), or divorce or separation from spouse (16.5%). Individuals from the lower-income quartiles reported a higher proportion (41.3%) of food insecurity than those from the higher-income quartiles (30.3%); however, the individuals from the higher-income quartiles reported a higher probability of job loss (43.1% vs 25.0%) or divorce (22.4% vs 11.7%) than those from the lower-income quartiles.

Nearly half (45.3%) of the individuals perceived the financial impact of anti-tuberculosis treatment to be serious or very serious. Individuals from the lower-income quartiles reported more serious perceived impacts than those from the higher-income quartiles (64.5% vs 39.5%, *P* < 0.001). The details are shown in Table 5.

**Table 5:** Proportions of tuberculosis-affected households reporting impoverishing mechanisms and social consequences

	Total	Income quartiles ^a^			
		Q1	Q2	Q3	Q4
Coping strategies					
Loan only	199 (45.8)	66 (63.5)	61 (54.0)	39 (35.8)	33 (30.3)
Sale of productive assets only	2 (0.5)	1 (1.0)	0 (0.0)	1 (0.9)	0 (0.0)
Loan and sale of productive assets	10 (2.3)	2 (1.9)	5 (4.4)	2 (1.8)	1 (0.9)
Any combination of the three above	211 (48.6)	69 (66.4)	66 (58.4)	42 (38.5)	34 (31.2)
Social consequences					
Food insecurity	149 (34.3)	43 (41.3)	37 (32.7)	36 (33.0)	33 (30.3)
Divorce or separation from spouse	56 (16.5)	9 (11.7)	12 (13.3)	16 (18.2)	19 (22.4)
Loss of job	156 (35.9)	26 (25.0)	37 (32.7)	46 (42.2)	47 (43.1)
Interruption of child’s schooling	10 (2.3)	1 (1.0)	6 (5.3)	2 (1.9)	1 (0.9)
Social exclusion	139 (31.9)	30 (28.8)	38 (33.6)	34 (31.2)	37 (33.9)
Self-reported influences					
No impact	37 (8.5)	9 (8.7)	6 (5.3)	6 (5.5)	16 (14.7)
Little impact	94 (21.6)	9 (8.7)	23 (20.4)	32 (29.4)	30 (27.5)
Moderate impact	107 (24.6)	19 (18.3)	39 (34.5)	29 (26.6)	20 (18.3)
Serious impact	114 (26.2)	35 (33.7)	25 (22.1)	27 (24.8)	27 (24.8)
Very serious impact	83 (19.1)	32 (30.8)	20 (17.7)	15 (13.8)	16 (14.7)

^a^Income quartiles are arranged from lower to higher (Q1 = lower; Q4 = higher). Abbreviations: *Q1* 1st Quartiles; *Q2* 2nd Quartiles; *Q3* 3rd Quartiles; *Q4* 4th Quartiles

## Discussion

Using a cross-sectional study of TB patients in NTP networks, this study showed that TB patients faced considerable payments and financial losses, even in the most developed areas in China. Nearly half of the costs occurred before treatment, and two-thirds of costs were due to direct medical costs. Several coping strategies, especially loans, were used by half of the patients. Additionally, one-third of the TB patients experienced food insecurity and social exclusion. Both economic consequences and social consequences for TB care varied across the different income quartiles, with a higher ratio of cost to household income and a higher proportion of loan and food insecurity in poorer households.

### Interpretation of key findings

#### OOP payments and their components

This study showed that the average OOP payment was USD 2389.5 for TB patients during the whole TB episode, which was equivalent to 27.4% of the annual household income. The proportion was higher than that in Pakistan (5.4%) [15] and lower than those in Ethiopia (152%) [21] and Nigeria (37%) [22].

Direct medical costs accounted for the majority of the costs in this population, inconsistent with other studies that showed a high proportion of non-medical costs and indirect costs [23, 24]. One reason for high medical costs may be due to diagnosis delay, which is supported by the fact that medical costs before TB treatment accounted for one-third of total costs, especially among the patients with the lowest household income (40.5%). Additionally, for the treatment period, medical costs in the intensive phase still were a dominant part of the costs incurred during treatment in this population. Under the free TB care policy, TB patients are only provided with a free chest X-ray, sputum smear test and first-line drugs in TB designated medical facilities in China. Second-line anti-tuberculosis drugs and liver protective drugs are two common sources of the patients’ direct medical costs in this period [25, 26], which is different than most TB endemic locations in the world. Meanwhile, most of the patients in this study were from rural areas, with their income mainly coming from farming or casual labour, or were economically inactive (housewife/unemployed, students and retirees). Therefore, TB care had little impact on their time loss [27].

In our study, cost distributions were unequal across the income quartiles; poor households spent a higher proportion of direct medical costs before TB treatment. This finding may be explained by their poor awareness of TB and seeking health care from informal care providers [28, 29].

#### Income equality in economic and social consequences

TB is a poverty-related disease, and it disproportionately affects the most economically disadvantaged stratum of society [8, 30, 31]. In our case, patients from the higher-income quartiles were more likely to have higher OOP payments than those from the lower-income quartiles; however, total OOP costs as a percentage of household income decreased with the household income level. The same results were reported in other studies that showed that poorer households had lower capacities to pay and that even low healthcare costs had heavy impacts on their household [15, 32]. Meanwhile, our results showed that irrespective of the household socioeconomic status, when TB occurred in a household, it plunged the household below the poverty line, as was found in another study [22]. However, the impoverishing impact of OOP health expenditures was higher in poorer households, which was consistent with previous studies and confirmed the ‘medical poverty trap’ situation where impoverishment was caused by paying for medical care [33, 34].

Regarding coping strategies and social consequences, income inequality also existed. Nearly half of TB patients adopted coping strategies to finance their health expenses, which was lower proportion than that previously reported in Nigeria (88.0%) and in Tajikistan (65.7%) [22, 35]; coping strategies were also more common among poor households. However, there were fewer social consequences, except food insecurity, in poor households. The reason needs to be clarified in future studies, especially with qualitative surveys.

### Policy implications

Medical costs constituted a major proportion of total costs and led to high costs of TB care, which suggest that the current free TB policy does not guarantee financial risk protection. Measures to reduce and compensate for patients’ direct medical costs are needed, including comprehensively expanding the benefit package for patients. Meanwhile, a large portion of the costs occurred before treatment started, which suggests that it is necessary to train health system staff in general hospitals to identify and refer TB patients to BMUs.

A greater economic and social burden in the lower income group suggests a need for government pro-poor programmes to provide protection specifically for poor households.

### Limitations

To avoid recall bias, the population in this study was TB patients who were currently being treated. However, this approach introduced a problem, as only the costs from the onset of symptom to the treatment status at the time of the interview were collected, and the costs from the TB phase from interview to the end of treatment needed to be estimated. Due to the regular frequency of doctor visits and the medicine doses at each treatment stage, it was reasonable to use the relationship between visit time and cost after adjusting for disease severity and socioeconomic status. However, the costs may fluctuate towards the end of treatment, for example, when a person’s TB illness improves or when a person regain employment, which would lead to bias from the cost estimation in the continuous phase.

## Conclusions

Under the current “free diagnosis and treatment” policy, TB patients still face high OOP costs with economic and social consequences. Medical and pretreatment costs constitute a major proportion of total costs throughout the whole TB process, which suggests that the current free service policy is not enough to protect TB patients. High inequity in economic and social consequences for poor households shows that providing targeted financial and social support for poor groups can effectively alleviate the economic burden experienced by households affected by TB.

## Data Availability

The dataset and codebook used in this study are available on request from the corresponding authors (liwang@ibms.pumc.edu.cn, zhanghui@chinacdc.cn).

## Abbreviations

CDC: Center for Disease Control
NCMS: New Cooperative Medical System
OOP: Out-of-pocket
TB: Tuberculosis
UEBMI: Urban Employee Basic Medical Insurance
URBMI: Urban Residents’ Basic Medical Insurance
